# Strategies to manage tree pest and disease outbreaks: a balancing act

**DOI:** 10.1186/s12862-023-02184-0

**Published:** 2023-12-06

**Authors:** Cecilia A. L. Dahlsjö

**Affiliations:** School of Geography and the Environment, University of Oxford, Oxford, UK

**Keywords:** Trees, Forests, Disease prevention and mitigation, Pests, Pathogens, Tree felling

## Abstract

Tree diseases are one of the major threats to forests worldwide. As the frequency and severity of disease outbreaks increase, effective prevention and mitigation strategies are urgently needed. Emerging methods are available to tackle this issue, however, trade-offs and potential ecological consequences should be considered for successful forest preservation.

## Main text

Tree pests and pathogens are a natural part of ecosystems and play an important role in forest dynamics. However, largely due to human actions, such as global trade and climate change, the severity and frequency of tree disease outbreaks have increased worldwide [1]. As threats to trees grow, there is an increasing need for effective disease prevention and mitigation strategies to preserve forests and their ecosystem services. Tree diseases have economic and ecological consequences due to their impact on timber quality and habitat dynamics. In Britain, ash dieback is expected to cost £15 billion over a 100 year period, although half of the cost is predicted in the first decade [2]. Ecologically, widespread tree diseases have cascading effects on ecosystems which influence everything from nutrient cycling and habitat structure to population dynamics and species behaviour [3]. Weakened trees will also increase branch and tree falls which may pose a risk to the public.

The global movement of plant materials causes a continuous introduction of non-native pests and pathogens to all corners of the world. Ash dieback (*Hymenoscyphus fraxineus*), a fungal disease brought to Europe from Asia by imported timber, is expected to have a 70% mortality rate across Europe [4]. However, tree diseases may also be caused by populations of native species. Changes in population dynamics due to climate change have turned some native species into pests, such as the spruce beetle (*Dendroctonus rufipennis*) and the aspen leaf miner (*Phyllocnistis populiella*) in the United States [1]. Threats can occur at short notice or be hard to detect before they cause unpreventable damage, such as those caused by microbial pathogens. Therefore, conducting disease mitigation in a timely manner to safeguard tree populations is a complex endeavour. Expectedly, resources tend to focus on diseases that pose the most severe economic and ecological threat.

As the Dutch philosopher Desiderius Erasmus said, “*Prevention is better than cure”.* While the saying is mainly used as an anecdote in healthcare, it is highly relevant for forest disease mitigation. Improved and better enforced biosecurity to detect, intercept, and prevent the spread of invasive pests and pathogens are needed to reduce the number of disease outbreaks. Governments are now beginning to wake up to biosecurity risks and are developing strategies to protect native tree species. For example, in 2023, the Department for Environment, Food and Rural Affairs (DEFRA) in partnership with the Forestry Commission and the Scottish and Welsh Governments published a new five year action to improve biosecurity and protect native tree species in Britain [5].

At the local scale, creating forest ecosystems that are resilient to tree diseases is both economically and ecologically beneficial due to the cost of damage repair and the time it takes for ecosystems to recover. Forests with mixed species and age composition often have the highest resilience against tree diseases as the distance between and susceptibility of trees affect infection and mortality rates [6, 7]. Species selection is therefore important to ensure that the trees are adapted to the local environment and not vulnerable to the same diseases. Tree diversity also reduces the financial burden of diminished timber quality and has a smaller ecological effect as trees grow into the available space caused by the disease [8]. The ecological impact does, however, depend on the ability of other species to maintain the ecosystem properties of the affected group including support for tree-associated organisms and nutrient cycling [9].

Passive surveillance and citizen science are being used to spot new pest and disease threats to trees. In Britain, Forest Research developed a web-based tool called TreeAlert to enable members of the public to report visible symptoms of ash dieback (canopy dieback) [10]. In Sweden, a citizen science programme called Rädda Asken (save the ash) (https://raddaasken.nu/) educates the public about ash dieback and encourages people to report the occurrence of healthy ash trees, particularly if they are surrounded by diseased trees. The latter helps to identify more tolerant and resistant ash trees that can be used in breeding programmes.

Tree diseases are often detected after symptoms are visible - particularly for microbial pathogens that are difficult to see with the naked eye. Detection may also be slow if pathogens are air or soilborne as they may take a long time to develop. Fuelled by recent technological advances, scientists are using increasingly sensitive and rapid molecular techniques, such as the analysis of environmental DNA, to detect tree pathogens [11]. For example, quantitative PCR has been successfully used during peak sporulation to examine the presence of airborne fungal pathogens. *H. fraxineus* and *Ceratocystis platani* (*Platanus* sp disease) have been detected 100 and 200 km from the closest symptomatic tree, respectively, using this method [11]. Such early detections enable effective disease mitigation before symptoms occur which is vital for successful long-term forest preservation.

Once disease outbreaks occur, appropriate management is needed to reduce their effect and spread. When symptomatic trees cause the public an unavoidable safety risk, they should be removed accordingly. For all other trees, however, the most effective disease management will likely vary depending on the vector type and whether timber quality is an important consideration. Removal of infected trees may be appropriate in commercial sites as the felled trees retain their timber quality while the introduced gaps can reduce disease spread [12]. Regular forest maintenance and removal of trees that are damaged or have been weakened by disease may reduce the chance of infection and subsequently the likelihood of severe outbreaks [13, 14].

However, tree felling may in some cases increase disease risk as organisms such as the pine weevil that causes damage to a wide range of both conifer and broadleaved trees, lay eggs in tree stumps [15]. Additionally, tree felling may not target the main cause of the disease which means that the symptoms can persist long after the symptomatic trees are gone, which is the case for root rot [6]. Pruning of infected branches may increase the lifespan of individual trees [16], although it is unlikely to have a substantial effect on disease spread and can increase the risk of infection through the exposed surfaces.

Habitat disturbance, caused by disease or tree fall, is a natural phenomenon that increases heterogeneity and fosters greater species diversity due to the formation of microhabitats and localised habitat succession. Trade-offs between ecological function and disease management must therefore be carefully considered on a case-by-case basis. If a disease outbreak has occurred, tree felling may delay but not prevent infection rates, while stump removal could reduce infection rates but may have a negative effect on specialist species [6]. Human activity, increased site access, and the deployment of machinery may cause unintended increases in disease spread due to the movement of pests and pathogens [6]. Tree felling must also be weighed against the importance of trees for biodiversity connectivity [6]. Standing dead trees play a major role in supporting biodiversity both directly by creating habitats for deadwood species and indirectly by providing shelter and food resources for birds and mammals [17]. Leaving diseased trees in situ will also enable monitoring of individuals that may be resistant to the disease. These individuals will promote natural resilience in the population and may be used in breeding programmes to produce more disease resilient forests for the future. Tree felling is also an expensive activity that adds to the economic cost of disease outbreaks that must be weighed against the cost of not taking action. It is therefore important to fully understand the potential effects of tree felling as it may not reduce disease risk and have unintended ecological consequences.

## Conclusions

Disease outbreaks cause habitat change which is a natural part of forest ecology. While tree felling plays a role in disease management, particularly in commercial sectors where timber quality is central, it is important to weigh up the trade-offs between the effectiveness of tree felling and the ecological consequences of such actions. Knowledge of the underlying causes of the disease will enable the most effective disease response. Creating functionally diverse forests that can better withstand outbreaks as well as managing ecosystems to reduce the occurrence of preventable diseases will be important for future tree disease mitigation. Governments and international companies are called upon to enhance their scrutiny of the consequences of global trade on the spread of infectious diseases. In the meantime, improved and enhanced biosecurity at a global scale will play a major role in reducing the threats of tree diseases worldwide.

## Data Availability

Not applicable.
